# Insights into the DNA-binding mechanism of a LytTR-type transcription regulator

**DOI:** 10.1042/BSR20160069

**Published:** 2016-04-27

**Authors:** Stefan Behr, Ralf Heermann, Kirsten Jung

**Affiliations:** * Munich Center for Integrated Protein Science at the Department of Microbiology, Ludwig-Maximilians-Universität München, 82152 Martinsried, Germany

**Keywords:** interaction map® (IM) analysis, protein–DNA interaction, pyruvate sensing, nutrient scavenging, response regulator YpdB, surface plasmon resonance (SPR) spectroscopy

## Abstract

Most bacterial response regulators (RRs) make contact with DNA through a recognition α-helix in their DNA-binding domains. An emerging class of RRs interacts with DNA via a relatively novel type of binding domain, called the LytTR domain, which is mainly composed of β-strands. YpdB belongs to this latter class, is part of a nutrient-sensing network in *Escherichia coli* and triggers expression of its only target gene, *yhjX*, in response to extracellular pyruvate. Expression of *yhjX* mainly occurs in the late exponential growth phase, and in a pulsed manner. Although the DNA-binding sites for YpdB are well defined, exactly how YpdB initiates pulsed gene expression has remained elusive. To address this question, we measured the binding kinetics of wild-type YpdB and the phosphomimetic variant YpdB-D53E to the *yhjX* promoter region (P*_yhjX_*) using surface plasmon resonance (SPR) spectroscopy combined with interaction map® (IM) analysis. Both YpdB and YpdB-D53E bound as monomers to the tandem-repeat sequences in the promoter, with YpdB-D53E displaying a higher maximal binding rate than YpdB. Furthermore, we identified a high-affinity (A-site) and a low-affinity binding site (B-site) within the *yhjX* promoter. Only YpdB-D53E utilizes an ‘AB-BA’ DNA-binding mechanism, involving sequential and cooperative promoter binding, and rapid, successive promoter clearance. We propose that response regulator phosphorylation, in combination with the cycle of cooperative DNA binding and rapid promoter clearance just described, can account for pulsed gene expression.

## Abstract (precis)

A combination of surface plasmon resonance (SPR) spectroscopy and interaction map® (IM) analysis was used to characterize binding of the LytTR-type response regulator YpdB to promoter DNA. YpdB follows an ‘AB-BA’ mechanism involving sequential and cooperative DNA binding followed by rapid successive promoter clearance.

## INTRODUCTION

Bacteria are equipped with specific signal transduction systems, which allow for optimal adaptation to changing environmental conditions. The most prominent bacterial signal transduction systems are of the histidine kinase/response regulator (HK/RR) type, also referred to as two-component systems. Upon stimulus perception, the HK autophosphorylates a highly conserved histidine, and subsequently transfers the phosphoryl group to a highly conserved aspartate in the cognate RR. The majority of RRs are DNA-binding proteins, which act as transcriptional regulators when phosphorylated [1]. The family of LytS/LytTR HK/RR systems is widespread among human and plant pathogenic bacteria, and controls production of a variety of virulence and virulence-associated factors. Among known LytS/LytTR-regulated pheno-types are toxin production in *Staphylococcus aureus* [2], natural competence in *Streptococcus pneumoniae* [3] and the biosynthesis of extracellular polysaccharides in *Pseudomonas aeruginosa* [4,5].

High-resolution structural analyses of LytTR-family members have revealed an uncommon DNA-binding domain. A conserved 10-stranded β-fold mediates DNA binding via three elongated β-sheets, whereas highly variable amino acids within the connecting loop-regions are responsible for binding specificity [6]. The DNA-binding sites of almost all LytTR RRs are similar in their overall structure, comprising two direct or inverse repeats (9–11 nucleotides in length) separated by 11–13 spacer nucleotides, but they do not share a common consensus binding sequence [7–9]. Moreover, the flanking DNA regions also seem to be important for binding of LytTR-type transcription factors [10–13]. The YpdA/YpdB system of *Escherichia coli*, a LytS/LytTR-type HK/RR system, has recently been investigated in more detail [13]. The HK YpdA responds to extracellular pyruvate and activates its cognate RR YpdB, which in turn induces *yhjX* expression [13]. The gene *yhjX* is the only direct target gene of YpdB and codes for a putative transport protein of the major facilitator superfamily. When *E. coli* is grown in peptone broth, pulsatile induction of *yhjX* is observed during the late exponential growth phase [14]. The gene product is assumed to contribute to nutrient scavenging before cells enter stationary phase [13]. The RR YpdB is composed of an N-terminal receiver domain, with a conserved aspartate at position 53, and a C-terminal LytTR effector domain with DNA-binding affinity. Replacement of aspartate 53 by glutamate (YpdB-D53E) results in constitutive *yhjX* expression, presumably because the variant mimics phospho-YpdB [13].

Although two studies have provided detailed insights into the structural properties of the LytTR domain of AgrA from *S. aureus* when bound to its cognate DNA sequence [6,15], details of the mechanism of transcriptional activation by LytTR-type transcriptional regulators are still obscure. Only Straume et al. [16] have reported cooperative DNA binding by PlnC to tandemly arranged sites in the *plnA* promoter. It nevertheless remains unclear how the LytTR-type transcription factor YpdB induces pulsed expression of *yhjX* [13]. In order to answer this question, our study used a combination of surface plasmon resonance (SPR) spectroscopy with interaction map® (IM) analyses to determine the binding kinetics of YpdB and its phosphomimetic variant YpdB-D53E to the tandem repeat sequences in the *yhjX* promoter.

## MATERIALS AND METHODS

### Protein purification and molecular biological techniques

For the purification of 6His-YpdB and 6His-YpdB-D53E, *E. coli* strain BL21(DE3) [17] was transformed with pBAD24-*ypdB* and pBAD24-*ypdB*-D53E respectively [13]. Overproduction of 6His-tagged proteins and subsequent purification by Ni-NTA-affinity chromatography was performed as described before [12], yielding approximately 95% pure protein as estimated by SDS-PAGE [18]. The oligomeric state of both proteins was analysed using size-exclusion chromatography on a calibrated Superdex 200 Increase GL HR10/300 column (GE Healthcare), equilibrated with 50 mM Tris/HCl pH 7.6, 10% (v/v) glycerol, 150 mM NaCl, 2 mM DTT.

The size of biotinylated DNA fragments used in the SPR measurements was adapted from earlier experiments initially characterizing the *yhjX* promoter [13] and resembles all protected nucleotides from DNaseI footprint experiments. To generate double-stranded biotinylated P*_yhjX_*, equimolar amounts of 5′-biotinylated oligonucleotides (Sigma–Aldrich) and their complementary non-biotinylated strands (flanked by four guanine-cytosine nucleotides) (Table 1) were first heated at 95°C for 5 min and subsequently annealed by slow cooling to room temperature. The additional four G-C flanking the promoter region were added to further stabilize the DNA fragment and are therefore referred to as clamp. Depending on the oligonucleotides used the motifs of interest in the biotinylated double-stranded DNAs carried purine-to-pyrimidine or pyrimidine-to-purine transversions.

**Table 1:** Oligonucleotides used for SPR experiments on a SA sensor chip Biotinylated [BTN] oligonucleotides of the native promoter as well as BTN-oligonucleotides with motif substitutions (lower case letters) were annealed, and the double stranded DNA was immobilized onto the surface of a SA sensor chip. As a control the promoter DNA fragment of P*_yjiY_* was used.

Name	5′ → 3′ sequence
[BTN] *yhjX* YpdB bs sense	[BTN]GGGGCGCGTCATTCATTCCTGAACTAAGGCATTTCATTCCGTTCTGATGGCATTTCATGCCGGGGG
*yhjX* YpdB bs antisense	CCCCCGGCATGAAATGCCATCAGAACGGAATGAAATGCCTTAGTTCAGGAATGAATGACGCGCCCC
[BTN] *yhjX* YpdB A bs sense	[BTN]GGGGCGCGTCATTCATTCCTGAACTAAttacgggacgTCCGTTCTGATGGCATTTCATGCCGGGGG
*yhjX* YpdB A bs antisense	CCCCCGGCATGAAATGCCATCAGAACGGAcgtcccgtaaTTAGTTCAGGAATGAATGACGCGCCCC
[BTN] *yhjX* YpdB B bs sense	[BTN]GGGGCGCGTCATTCATTCCTGAACTAAGGCATTTCATTCCGTTCTGATttacgggacgGCCGGGGG
*yhjX* YpdB B bs antisense	CCCCCGGCcgtcccgtaaATCAGAACGGAATGAAATGCCTTAGTTCAGGAATGAATGACGCGCCCC
[BTN] *yjiY* YehT bs sense	[BTN]GGGGCCTTTGCCGCTCAACCGCAAAACTGACCGCTTACATCCCTAAAATAACCACTCAGTTAGGGG
*yjiY* YehT bs antisense	CCCCTAACTGAGTGGTTATTTTAGGGATGTAAGCGGTCAGTTTTGCGGTTGAGCGGCAAAGGCCCC

### Surface plasmon resonance spectroscopy

SPR assays were performed in a Biacore T200 using carboxymethyl dextran sensor chips pre-coated with streptavidin (SA Sensor Chip Series S). All experiments were carried out at a constant temperature of 25°C using HBS-EP [10 mM HEPES pH 7.4, 150 mM NaCl, 3 mM EDTA, 0.005% (v/v) detergent P20] as running buffer. Before immobilizing the DNA fragments, the chips were equilibrated by three injections of 1 M NaCl/50 mM NaOH applied at a flow rate of 10 μl/min. Then the respective double-stranded biotinylated DNA fragment (10 nM) was injected at a flow rate of 10 μl/min for a total contact time of 420 s. The chips were then washed by injecting 1 M NaCl/50 mM NaOH/50% (v/v) propan-2-ol. Approximately 100–200 RU (response units) of the relevant DNA fragment was bound per flow cell. Analyses of the kinetics of interaction of YpdB and YpdB-D53E with the various DNA fragments were performed at a flow rate of 30 μl/min in HBS-EP buffer at 25°C. Various concentrations of the proteins (1–50 nM), dissolved in HBS-EP buffer, were passed over the flow cells for 180 s, and the complexes formed were allowed to dissociate for 300 s before the next cycle started. After each cycle, the surface was regenerated by injection of 2.5 M NaCl for 30 s, followed by 0.5% (w/v) SDS for 60 s, at a flow rate of 30 μl/min. All experiments were performed at 25°C. Sensorgrams were recorded using Biacore T200 Control Software 1.0 and analysed with Biacore T200 Evaluation Software 1.0. The surface of flow cell 1 was coated with a control DNA (P_*yjiY*_ DNA, no binding) and used to obtain blank sensorgrams for subtraction of the bulk refractive index background. The referenced sensorgrams were normalized to a baseline of 0. Peaks in the sensorgrams at the beginning and the end of the injection are due to the run-time difference between the flow cells for each chip.

Concentration-free calibration analysis (CFCA) was performed using a standard 5 μM solution of purified YpdB-D53E (calculated from absorbance-based determination of protein concentration using a molar absorption coefficient for YpdB of 3.24×10^5^ M^−1^·cm^−1^), which was diluted stepwise (1:2, 1:5, 1:10 and 1:20). Each protein dilution was injected twice, first at 5 μl/min, then at 100 μl/min. P*_yhjX_* DNA was used for YpdB-D53E binding in the active flow cell, and P*_yjiY_* DNA (control DNA) in the reference cell. CFCA basically relies on mass transport, which is a diffusion phenomenon that describes the movement of molecules between the solution and the surface. The CFCA therefore relies on the measurement of the observed binding rate during sample injection under partially or completely mass transport-limited conditions. In general, the initial binding rate (d*R*/d*t*), which is dependent on the diffusion constant of the protein, is measured at two different flow rates. The diffusion coefficient of YpdB-D53E was calculated by the Biacore diffusion constant calculator and converter webtool (https://www.biacore.com/lifesciences/Application_Support/online_support/Diffusion_Coefficient_Calculator/index.html), based on the assumption that the protein is globular in shape. In this way, the diffusion coefficient of YpdB-D53E was determined to be *D*=9.94×10^−11^ m^2^·s^−1^. The initial rates for those dilutions that differed by a factor of at least 1.5 were utilized to calculate the ‘active’ concentration, which was determined as 5×10^−8^ M (1% of the absorbance-based measured protein concentration) for YpdB-D53E. The active protein concentration was then used for calculation of the kinetic binding constants.

### Interaction map® analysis

IM calculations were performed on the Ridgeview Diagnostic Server (Ridgeview Diagnostics, Uppsala, Sweden). For this purpose, the SPR sensorgrams were exported from the Biacore T200 Evaluation Software 1.0 as *.txt files and imported into TraceDrawer Software 1.5 (Ridgeview Instruments). IM files were created using the IM tool within the software, generating files that were sent via e-mail to the server (im@ridgeviewdiagnostics.com), where the IM calculations were performed [19]. The resulting files were then evaluated for spots in the TraceDrawer 1.5 Software, and the IM spots were quantified.

## RESULTS AND DISCUSSION

### Purified 6His-YpdB and 6His-YpdB-D53E are monomeric in solution

YpdB-mediated expression of *yhjX* in *E. coli* cells grown in LB medium follows upon the accumulation of extracellular pyruvate and results in a pulse-shaped burst of transcription upon entry into stationary phase. Replacement of the highly conserved aspartate residue at position 53 within the receiver domain of the RR by glutamate (YpdB-D53E) causes *yhjX* expression to become constitutive and independent of growth phase [13]. Aspartate-to-glutamate substitutions have been shown to simulate phosphorylation in several RR classes [20], and this property, which often increase their DNA-binding affinities [7–9], has allowed the characterization of the active states of RRs [21].

In the present study, we have compared the properties of the constitutively active YpdB-D53E with those of the inactive wild-type protein to gain insights into the mechanism of promoter activation by this LytTR-type transcriptional activator.

**Figure 1:**
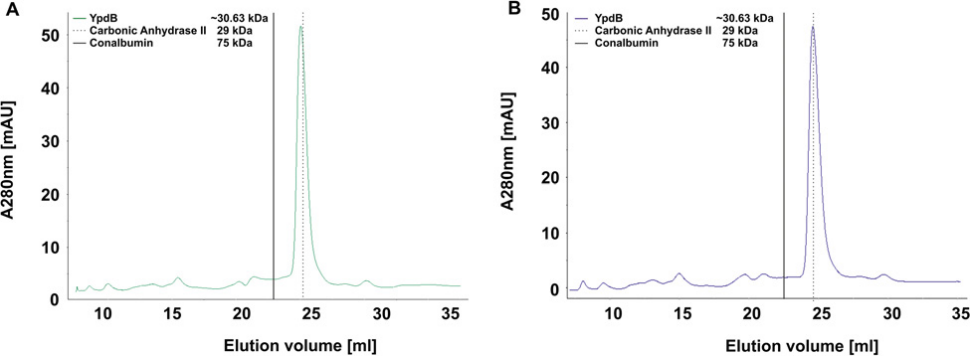
Absorbance spectra obtained after size exclusion chromatography of purified 6His-YpdB and 6His-YpdB-D53E After affinity chromatography via an N-terminal 6His-tag, the oligomeric state of (**A**) YpdB (green) and (**B**) YpdB-D53E (blue) was analysed by size-exclusion chromatography using a calibrated Superdex 200 Increase GL HR10/300 column. The solid line represents the retention volume of 75 kDa protein conalbumin whereas the dotted line refers to 29 kDa protein carbonic anhydrase II, both applied in the same buffer used for 6His-YpdB/6His-YpdB-D53E purification.

For *in vitro* studies, 6His-YpdB and 6His-YpdB-D53E were overproduced and purified by Ni-NTA-affinity chromatography as described before. Subsequent size-exclusion chromatography showed that both species (with an estimated molecular mass of 30.6 kDa) are exclusively monomeric in solution (Figures 1A and 1B).

### YpdB-D53E binds to the P*_yhjX_* promoter with high affinity

In the next step we determined the kinetics of binding of YpdB and YpdB-D53E to the native promoter (P*_yhjX_*) using SPR spectroscopy.

**Figure 2:**
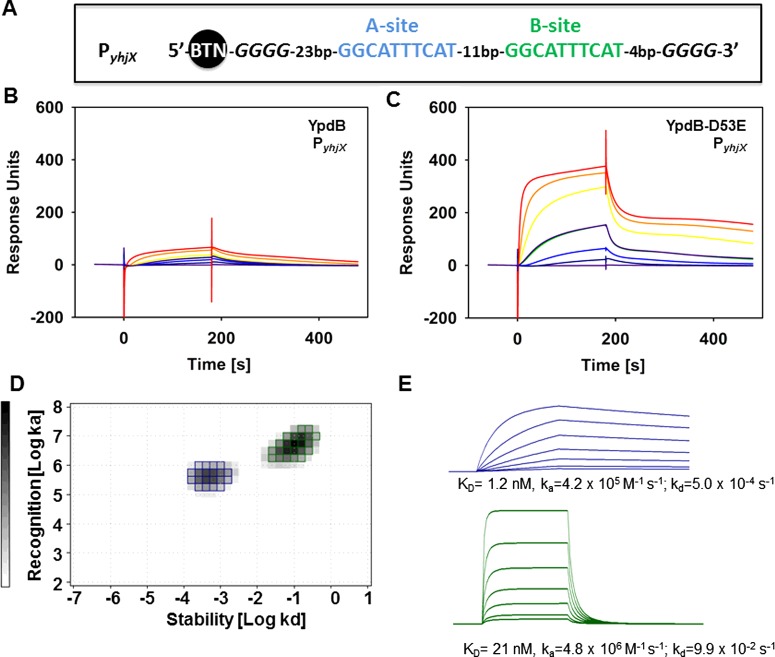
Binding of YpdB and YpdB-D53E to the promoter region of *yhjX* (P*_yhjX_*) (**A**) Schematic presentation of the binding sites A (blue) and B (green) within the biotinylated *yhjX* promoter fragment used in the consecutive SPR analyses. (**B** and **C**) The biotin-labelled DNA fragment comprising P*_yhjX_* was captured onto a SA sensor chip, and solutions containing purified YpdB at concentrations of 1 nM (violet line), 2.5 nM (blue line), 5 nM [dark blue and green line (internal reference)], 10 nM, 20 nM (orange line) and 50 nM (red line) (**B**) or YpdB-D53E (**C**) were passed over the chip. (**D**) IM analyses of the YpdB-D53E-P*_yhjX_* interaction. The green and blue spots both represent interactions of YpdB-D53E with the P*_yhjX_* DNA. (**E**) Sensorgrams were inferred for each specific *K*_D_ value calculated from the IM analyses. The blue sensorgram corresponds to the blue spot and the green sensorgram to the green spot from ON/OFF rate map. The calculated affinities, as well as the ON/OFF rates, are indicated below the sensorgrams.

The corresponding 5′-biotinylated double-stranded DNA (Figure 2A) was immobilized onto an SA Sensor Chip, and increasing concentrations (1–50 nM) of YpdB or YpdB-D53E were passed over the chip surface for 180 s (association), followed by a 300 s dissociation phase. We found that the maximal binding capacity (*R*_max_ RU) of YpdB-D53E to P*_yhjX_* (Figure 2C) was approxi-mately 8-fold higher than that of wild-type YpdB (Figure 2B). In addition, the sensorgrams differ in their overall association rates, which are significantly higher for YpdB-D53E than for YpdB to P*_yhjX_*.

Furthermore, simple affinity calculations based on maximal binding rates (excluding ON and OFF rates) would result in an overall affinity of YpdB-D53E for P*_yhjX_* of 10 nM. This value is approximately 10 times higher than the *K*_D_ value previously determined from gel-retardation experiments [13]. This discrepancy can be explained by the fact that the new calculation is based on the concentration of ‘active’ YpdB-D53E (as determined by CFCA) (for details please see Materials and Methods) and not on the total protein concentration.

### Binding of YpdB-D53E to the DNA is complex

A more precise evaluation of the YpdB-D53E binding curves revealed that none of the sensorgrams for the phosphomimetic RR followed a hyperbolic curve, which is expected for 1:1 binding events. This result indicates that the interaction of YpdB-D53E with its binding sites in P*_yhjX_* does not reflect equal binding of the RR to each single site, but supports the possibility of more complex binding interactions [16]. To calculate reliable binding constants and kinetic parameters, a computational approach was employed to evaluate the sensorgrams. In this approach, the experimental SPR curves are considered as the sum of individual binding curves, each representing a monovalent interaction [22]. Consequently, we generated a so-called interaction map® from the YpdB-D53E sensorgrams in order to determine and quantify the individual binding events represented by the SPR curves. In brief, the algorithm behind IM splits an experimental SPR data set into se-veral theoretically 1:1 binding curves, which need to be summed up to match the initially observed experimental data. By plotting the unique combination of the association rate *k*_a_ and dissociation rate *k*_d_ within a 2D distribution (surface plot with the function of *k*_a_ and *k*_d_), it is possible to display heterogeneous binding data as weight peaks, in which each peak corresponds to one component that contributes to the cumulative binding curve [19]. Fused peaks within this IM plot represent interactions with similar ON and OFF rates and can be taken into account with a peak weight proportion of more than 10%, whereas peaks below 10% most likely represent bulk effects. Based on the SPR sensorgrams for YpdB-D53E, IM analyses identified two clearly separated peaks (Figure 2D). The first peak (blue), with a peak weight of 33.5% (Figure 2D), displays an interaction with an average ON rate of 4.2×10^5^ M^−1^ · s^−1^ and an OFF rate of 5.0×10^−4^ s^−1^. Referred to a monovalent binding curve (Figure 2E, upper panel) it results in an overall affinity of 1.2 nM. The second peak (green), with a peak weight of 51.6% (Figure 2D), is characterized by a 10-fold higher ON rate, whereas the OFF rate for this interaction was determined to be 9.9×10^−2^ s^−1^, which is even 50-fold higher compared with the interaction derived from the first peak (blue). The monovalent binding curve of this interaction (green) results in a *K*_D_ of 21 nM (Figure 2E, lower panel).

In summary, the two YpdB-binding sites in P*_yhjX_* displayed the following features: One is characterized by a high affinity and has slow ON and slow OFF rates, whereas the second has a 10-fold lower affinity, with fast ON and fast OFF rates.

### Binding of YpdB-D53E to site B requires prior occupation of site A

**Figure 3:**
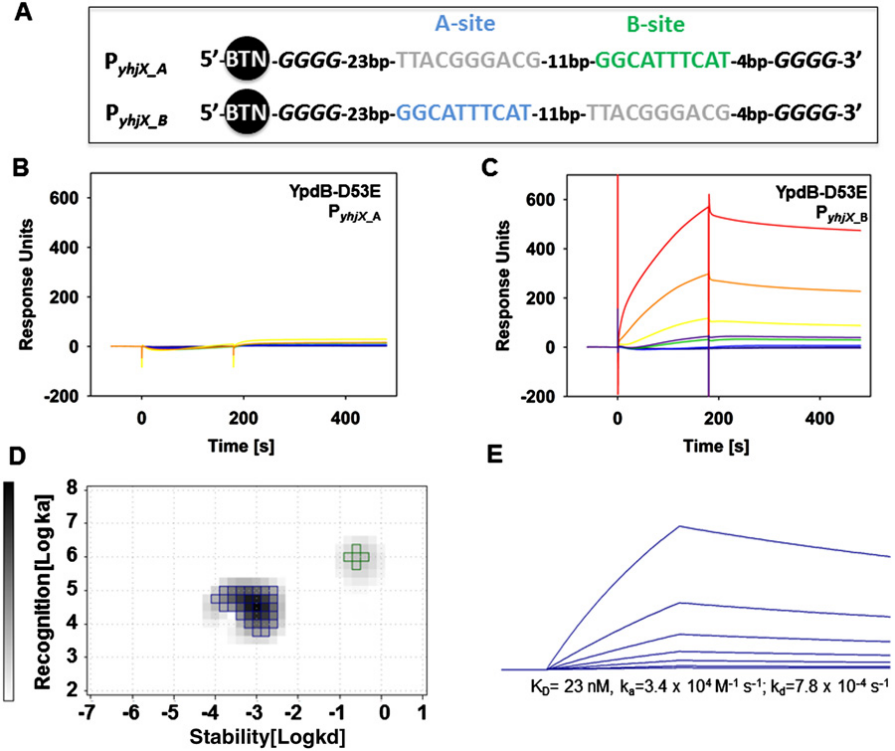
Binding of YpdB-D53E to the P*_yhjX_*__A_ and P*_yhjX_*__B_ (**A**) Schematic presentation of the two biotinylated *yhjX* promoter fragments carrying substitutions within binding site A or binding site B, respectively. (**B**, and **C**), SPR analyses. Biotin-labelled DNA fragments of the *yhjX* promoter region carry substitutions that inactivate site A (P*_yhjX_*__A_) (**B**) or site B (P*_yhjX_*__B_) (**C**), respectively. These fragments were captured onto SA sensor chips, and solutions of purified YpdB-D53E at concentrations of 1 nM (violet line), 2.5 nM (blue line), 5 nM [dark blue and green line (internal reference)], 10 nM (yellow line), 20 nM (orange line) and 50 nM (red line) were passed over the chip. (**D**) IM analyses of the YpdB-D53E-P*_yhjX_*__B_ interaction. The blue spot represents the YpdB-D53E interaction with the P*_yhjX_*__B_. The green spot with peak weight of <5% does not correspond to a binding event (see text). (**E**) Calculated sensorgram for the interaction of YpdB-D53E with P*_yhjX_*__B_. The calculated affinity, as well as the ON and OFF rates, are indicated below the sensorgram.

In order to probe these two interaction modes further, we examined the two YpdB-binding sites in more detail. YpdB binds to a well characterized direct repeat of the nucleotide motif GGCATTTCAT in P*_yhjX_* [13], hereinafter referred to as sites A and B (Figure 2A). We therefore generated two biotinylated DNA fragments in which either the A- (P*_yhjX_*__A_) or the B-site (P*_yhjX_*__B_) was inactivated by introducing transversion mutations (purines to pyrimidines and *vice versa*) (Figure 3A).

Then DNA fragments corresponding to P*_yhjX_*__A_ and P*_yhjX_*__B_, respectively, were captured onto an SA sensor chip, and SPR experiments using different concentrations of YpdB-D53E were performed as described above. Unexpectedly, we found that bin-ding of YpdB-D53E was completely abolished when site A was inactivated (Figure 3B). In contrast, YpdB-D53E was able to bind normally to P*_yhjX_*__B_, in which binding site B is mutated (Figure 3C). Moreover, in contrast with the binding of YpdB-D53E to the intact P*_yhjX_*, the sensorgrams for P*_yhjX_*__B_ recorded a single interaction event (Figure 3C). Calculation of the corresponding IM confirmed this observation (blue peak–weight of 68.2%) (Figure 3D). The additional small peak depicted in green with a peak weight of only 3.5% did not reflect a defined binding event, and was assumed to correspond to bulk effects. The ON (3.4×10^4^ M^−1^·s^−1^) and OFF (7.8×10^−4^ s^−1^) rates correspond to an affinity constant of 23 nM (Figure 3E). Compared with P*_yhjX_* the ON rate for YpdB-D53E binding to P*_yhjX_B_* was 10 times lower, whereas the OFF rate remained unaffected. Based on this observation the binding kinetics from the calculated binding curves of YpdB-D53E binding to P*_yhjX_* (Figure 2E) is in very good agreement with the monovalent interaction described by the blue peak, which is reflected in the upper panel of Figure 2E. Furthermore, we observed that the calculated peak in the IM peak (Figure 3D) was less sharply defined (see Figure 2D). This points to a broader range of variation for *k*_a_ and *k*_d_ values, which suggests that the YpdB-D53E-P*_yhjX_*__B_ interaction is less stable or might be affected by additional unspecific complex formation in the absence of the second binding site. Another explanation might be a disarrangement of the RR molecules that is otherwise prevented by the presence of an intact B-site. We therefore hypothesize that efficient binding of a second YpdB-D53E molecule to site B enhances protein–DNA interaction at site A by an increased overall stability.

### The ‘AB-BA’ binding model

In general, activation and subsequent dimerization of RR molecules is the predominant mode of action utilized for bacterial signal transduction by two-component systems [23]. Perception of the relevant signal induces the phosphorylation of a highly conserved aspartate in the receiver domain of the RR, which induces conformational changes and in turn results in activation of the protein. The relationship between activation and dimerization has been described for several RRs, such as VraR from *S. aureus* [24] and PhoB from *E. coli* [25]. Furthermore, structural studies of isolated receiver-domain variants of the LytTR-type RR ComE from *S. pneumoniae* have demonstrated that activation is tightly linked to the monomer-to-dimer transition [26].

We found that both 6His-YpdB and its constitutively active variant 6His-YpdB-D53E are monomeric in solution. The activation of YpdB, which is mimicked by the aspartate-to-glutamate substitution at position 53, enhances the protein's affinity for the P*_yhjX_* promoter region and the interaction follows bipartite binding kinetics. This suggests that phosphorylation of YpdB is not only important for initial DNA binding but also facilitates gene expression by enabling dimerization of two activated RR molecules on the DNA surface.

**Figure 4:**
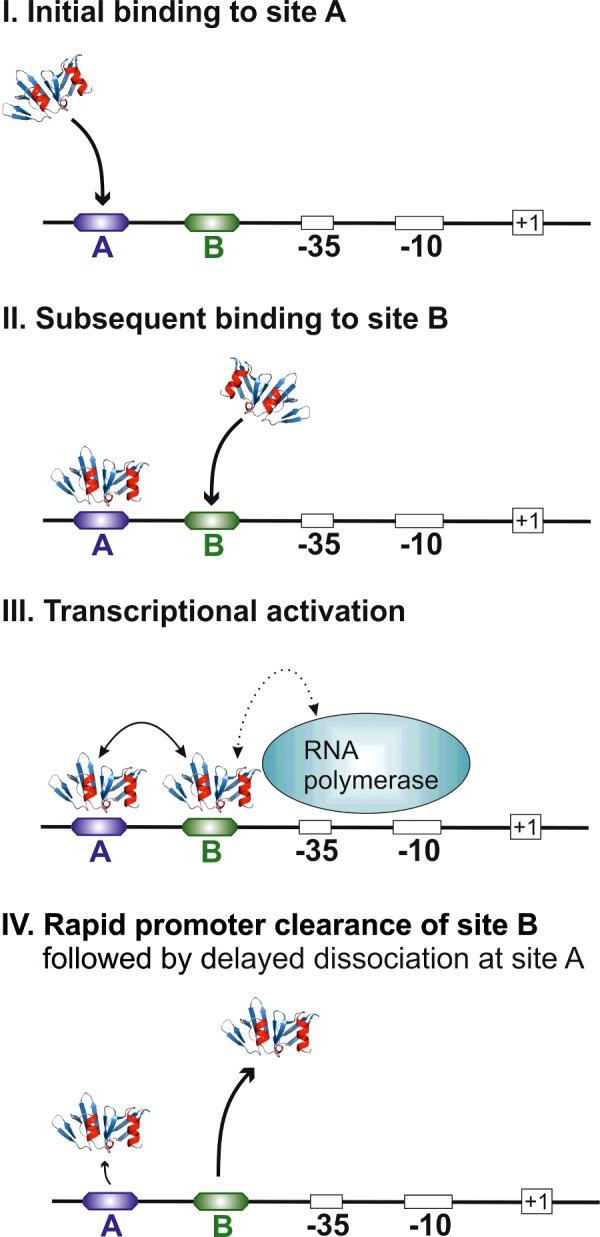
Schematic depiction of the ‘AB-BA’ mechanism of binding of activated YpdB (YpdB-D53E) to the *yhjX* promoter The RR is represented by the structural model of its DNA-binding domain derived by homology with known crystal structures. Initial binding of activated YpdB to *yhjX* promoter DNA occurs at site A. Subsequently, a second activated YpdB molecule occupies site B, and enables RNA polymerase recruitment and thus *yhjX* transcription. Occupation of site B is only transient, and initiates rapid and sequential promoter clearance.

Importantly, YpdB-D53E was completely unable to bind to P*_yhjX_* when site A was inactivated, whereas the protein could still bind to P*_yhjX_* when site B was mutated. These results suggest a two-step binding process, in which a molecule of YpdB-D53E must first bind to site A and, in so doing, stimulates binding of a second molecule to site B (Figure 4).

Furthermore, the decrease observed in the ON rate for binding of YpdB-D53E at the A-site when the B-site is inactivated indicates that two bound YpdB-D53E molecules stabilize each other. Thus, this two-step activation might serve to trigger conformational changes in the DNA structure. Structural studies of the LytTR-binding domain of AgrA from *S. aureus* have revealed strong bending of the DNA upon binding of two RR molecules [6]. Interestingly, DNA analyses based on the oligonucleotide sequence used for SPR experiments (Table 1) predicted an average curvature for the A-site of between 4 and 6°, whereas the B-site curvature ranged from 8 and 11° [27]. These values already point to clear structural differences between the two sequence motifs, obviously due to flanking nucleotides, which have been shown to influence expression *in vivo* as well [13]. Furthermore, binding of the second RR molecule could be stabilized by intermolecular protein interactions, as has been shown for the RR KdpE [28]. Besides DNA bending, binding of the second RR to the B-site will also provide the required protein interface for recruitment of RNA polymerase. After the induction of *yhjX* expression, promoter clearance is initiated by rapid dissociation of the (second) RR from the B-site. This should effectively prevent (excessive) gene expression and might serve, together with a slower dissociation of the first RR from the A-site, as a tight control mechanism to generate a pulsed or switch-like expression pattern [29].

In light of the sequential nature of the binding and clearance of activated YpdB to and from the A- and B-sites within P*_yhjX_*, we refer to this mode of operation as the ‘AB-BA’ mechanism (Figure 4).

YpdB belongs to the LytTR family of RRs, and the promoter motifs of their respective target genes are similar to those of the *yhjX* promoter, in that they all contain A- and B-sites of comparable lengths, which are separated by a similar distance. One can therefore postulate that ‘AB-BA’ might well be a general mechanism used by LytTR-type RRs to induce pulsed expression of genes that are particularly important for tightly regulated processes, such as toxin production or pathogenicity.

## Abbreviations

BTN: biotin
CFCA: concentration-free calibration analysis
HK/RR: histidine kinase/response regulator
IM: interaction map®
*k*_a_: association rate constant
k*_d_*: dissociation rate constant
*K*_D_: equilibrium dissociation constant (steady-state affinity)
P*_yhjX_*: promoter of the gene *yhjX*
RU: response units
SPR: surface plasmon resonance
